# How do social media use, gaming frequency, and internalizing symptoms predict each other over time in early-to-middle adolescence?

**DOI:** 10.1093/pubmed/fdaf150

**Published:** 2025-12-05

**Authors:** Qiqi Cheng, Margarita Panayiotou, Turi Reiten Finserås, Amanda Iselin Olesen Andersen, Neil Humphrey

**Affiliations:** Manchester Institute of Education, The University of Manchester, Oxford Rd, Manchester M13 9PL, UK; Manchester Institute of Education, The University of Manchester, Oxford Rd, Manchester M13 9PL, UK; Department of Health Promotion, Norwegian Institute of Public Health, Zander Kaaesgate 7, Bergen, 5015, Norway; Department of Health Promotion, Norwegian Institute of Public Health, Zander Kaaesgate 7, Bergen, 5015, Norway; Manchester Institute of Education, The University of Manchester, Oxford Rd, Manchester M13 9PL, UK

**Keywords:** mental health, social media, young people

## Abstract

**Background:**

The effects of adolescent digital technology use (e.g. social media, gaming) on their mental health are a major public health concern, but existing evidence is of mixed quality and findings have been inconclusive.

**Methods:**

Separating within-person effects from between-person effects, a random-intercept cross-lagged panel model was applied to three annual waves of data (T1, T2, T3) on social media use, gaming, and internalizing symptoms among N = 25 629 adolescents (51% girls, average age 12 years, 7 months (SD = 3.58 months) at baseline) in Greater Manchester, England.

**Results:**

Longitudinal relationships varied by gender, such that more frequent gaming at T2 predicted less time spent on social media use at T3 in girls (but not boys), and more frequent internalizing symptoms at T2 predicted reductions in gaming frequency at T3 in boys (but not girls). There was no evidence that time spent on social media or gaming frequency predicted later internalizing symptoms among girls or boys. Sensitivity analyses that distinguished active versus passive social media use replicated these findings.

**Conclusions:**

The findings of this study do not support the widely held view that adolescent technology use is a major causal factor in their mental health difficulties.

## Introduction

Over the last three decades, the prevalence of mental health difficulties (MHDs) among adolescents has steadily increased.^1^ Currently, about 22.6% of 11–16-year-olds have a probable mental disorder, with a further 12% reporting subthreshold symptoms.^2^ Internalizing difficulties (e.g. anxiety, depression) are the most prevalent in this phase of adolescence,^2^ and the peak age at onset in lifetime cases of such difficulties is 14.5 years,^3^ for which girls are disproportionately affected.^4^ There is a widespread view that adolescent digital technology use is the major underpinning causal factor.^5^ This study examines this claim, focusing specifically on how social media use, gaming frequency, and internalizing symptoms predict each other over time in early-to-middle adolescence.

Our starting point is a basic question: why do people engage with social media and gaming? The ‘uses and gratifications’ theory proposes that they actively choose media content to satisfy their specific needs and desires, as opposed to passively absorbing it.^6^ It recognizes that individuals seek different gratifications from the media with which they engage. The specific needs and gratifications sought are shaped by our characteristics, values, and beliefs and are influenced by social and cultural norms and expectations.^6^ In adolescence, peer relationships and identity formation are paramount, and these needs may motivate social media use; similarly, entertainment and social interaction needs may prompt engagement with gaming.^7^

Working from the uses and gratifications theoretical perspective, we would not necessarily anticipate adolescent digital technology use to predict mental health problems, and indeed, the evidence appears mixed at best. Several meta-analyses report on the association between adolescent social media use and mental health^8–11^ but vary in their conclusions. For example, one concluded that ‘increased social media use is associated with a range of negative mental health outcomes in adolescence’^10^ while another, published in the same year, was titled, ‘There is no evidence that time spent on social media is correlated with adolescent mental health problems’.^9^ It is also important to note that even when associations are found, the reported effect sizes in this body of literature are often modest, and a reliance in many meta-analyses on bivariate correlations, rather than controlled regression coefficients, may inflate estimates, suggesting true underlying effects could be even smaller.^12^^,^^13^ Expectancy effects and researcher positioning may play a role in this inconsistent interpretation.^8^ The potential negative effects of social media are also disproportionately focused upon, with potential benefits, such as social connection, sharing experiences with friends, and engaging in self-expression^14^^,^^15^ less frequently explored.

The extent to which video games impact adolescent mental health is also hotly debated.^16^ Early work focused on proposed social learning processes through which exposure to violent content in games might initiate aggressive behaviour among youth,^16^ as well as how increased gaming time correlated with more gaming-related problems.^17^ While there has been concern that some individuals may present with pathological patterns of video game use that interfere with life functioning,^18^ much of the research has found that it is possible to engage in extensive gaming without negative consequences.^19^^,^^20^ In fact, one meta-analysis reported that the negative impact of video gaming was minimal^16^ and indeed, a growing body of research has begun to recognize its potential cognitive, social, emotional, and motivational benefits.^21^

Contemporary research in this space emphasizes how social media and gaming might contribute to internalizing symptoms, but it is equally important to consider the converse effect. For instance, adolescents with symptoms of anxiety or depression might turn to social media for reassurance-seeking or mood regulation^22^^,^^23^ or engage in gaming to distract themselves from emotional distress.^24^

Despite a wealth of research on this topic, the evidence base is currently limited in several important respects. These include primarily cross-sectional work that does not warrant causal conclusions;^8^ use of small and homogeneous samples;^8^ failure to control for confounding factors (e.g. gender);^16^ and, in the case of social media research, a predominant focus on total time spent as opposed to ‘how’ that time is used.^25^ Finally, with a handful of exceptions,^26–29^ research to date has also not distinguished between-person (i.e. stable differences between individuals) from within-person (i.e. situational changes within individuals) effects. This is critical because failure to do so can lead to erroneous conclusions regarding the presence, predominance, and sign of causal influences.^30^

This study addresses the above issues and takes an important step in furthering our understanding of these relationships by using a longitudinal design, a very large and heterogeneous sample, inclusion of sensitivity analyses that distinguish active versus passive social media use, and application of the random intercept cross-lagged panel model (RI-CLPM) to enable separation of between-person from within-person effects. We note, however, a key limitation in the literature that we are unable to address in our secondary data analysis. Social media and gaming in the current study are both self-reported and thus do not capture an objective assessment nor a nuanced reflection of these constructs.^31^

Our motivating research question is how do social media use, gaming frequency, and internalizing symptoms predict each other over time in early-to-middle adolescence? Given the clear division in the extant literature and the unerring focus on this issue in the public arena, a study characterized by the methodological strengths outlined above offers a timely and important contribution to knowledge.

## Method

### Design and participants

Secondary analysis of the longitudinal cohort of the #BeeWell dataset^32^ was undertaken (ethical approval: 2021-11133-18965). A cross-lag panel design was used, drawing on three annual data points (T1, autumn 2021; T2, autumn 2022; T3, autumn 2023) for the three focal variables (social media use, gaming frequency, and internalizing symptoms). A ‘drop in’ approach was utilized to optimize sample size, including any participant with at least one data point.^33^ Those with missing values in covariates or missing all measures used in the analysis (N = 2 620) were excluded. A final sample of  N = 25 629 observations was utilized in the main analytical model, with a mean age of 12 years and 7 months (SD = 3.58 months), including 17% with special educational needs, 29% eligible for free school meals, and 34% from minoritized ethnic groups.

### Measures

Descriptive information about the measures is presented in Table 1.

**Table 1:** Means, standard deviations, and correlations between variables for each group.

Construct	SMU_T1	SMU_T2	SMU_T3	GAM_T1	GAM_T2	GAM_T3	INT_T1	INT_T2	INT_T3
Girls									
SMU_T1	1								
SMU_T2	0.567^***^	1							
SMU_T3	0.460^***^	0.561^***^	1						
GAM_T1	0.075^***^	0.062^***^	0.068^***^	1					
GAM_T2	0.039^**^	0.053^***^	0.018	0.454^***^	1				
GAM_T3	0.051^***^	0.048^***^	0.043^***^	0.394^***^	0.482^***^	1			
INT_T1	0.238^***^	0.163^***^	0.144^***^	0.046^***^	0.049^**^	0.051^***^	1		
INT_T2	0.162^***^	0.186^***^	0.145^***^	0.066^***^	0.048^**^	0.050^**^	0.633^***^	1	
INT_T3	0.129^***^	0.139^***^	0.157^***^	0.049^**^	0.049^**^	0.054^***^	0.485^***^	0.579^***^	1
Means	4.75	5.16	5.15	3.05	2.81	2.72	0.80	0.82	0.77
Std. Dev.	2.45	2.30	2.24	1.22	1.26	1.27	0.42	0.43	0.44
Boys									
SMU_T1	1								
SMU_T2	0.467^***^	1							
SMU_T3	0.390^***^	0.469^***^	1						
GAM_T1	0.072^***^	0.069^***^	0.075^***^	1					
GAM_T2	0.022	0.090^***^	0.062^***^	0.299^***^	1				
GAM_T3	0.014	0.031	0.065^***^	0.208^***^	0.299^***^	1			
INT_T1	0.101^***^	0.087^***^	0.067^***^	−0.066^***^	−0.047^*^	−0.021	1		
INT_T2	0.044^**^	0.100^***^	0.069^***^	−0.027	−0.100^***^	−0.090^***^	0.573^***^	1	
INT_T3	0.050^**^	0.058^***^	0.096^***^	−0.028	−0.054^*^	−0.163^***^	0.410^***^	0.491^***^	1
Latent Means	3.92	4.19	4.29	3.79	3.74	3.70	0.53	0.50	0.48
Latent Std. Dev.	2.49	2.34	2.29	0.67	0.73	0.78	0.36	0.39	0.42

Note. Results are based on the effects-coded method of identification with the scalar invariant across time and metric invariant across gender. SMU, social media use; GAM, gaming frequency; INT, internalizing symptoms; T1, 2, 3 = Time 1, 2, 3.

^*^ *p* < .05. ^**^p < .01. ^***^p < .001.

#### Social media use

A single item adapted from the Millennium Cohort Study survey^34^ was used: ‘On a normal weekday during term time, how much time do you spend on social media? For example, sites or apps like TikTok, Instagram, and Snapchat’. Participants selected responses in hourly increments from 0 to 7+. For those reporting >0 hours, conditional follow-up items asked them to estimate the proportion of time spent engaging in active (‘How much of the time noted above do you spend doing things like chatting with others, and posting stories, pictures and videos?’) versus passive (‘How much of the time noted above do you spend doing things like browsing feeds, profiles or scrolling through photos and stories?’) activities, with the survey set such that responses had to total 100%.

#### Gaming frequency

A single-item scale adapted from the Millennium Cohort Study survey^34^ was used: ‘How often do you play games on a computer or games console, such as Nintendo Switch, Xbox, or PlayStation?’ Response options were: Most days, At least once a week, At least once a month, Several times a year, Once a year or less, and Never or almost never. These responses were recoded into a four-point scale: 1 = Never or almost never/once a year or less (rarely); 2 = Several times a year (occasionally); 3 = At least once a month (sometimes); and 4 = Most days/at least once a week (often).

#### Internalizing symptoms

The 10-item emotional difficulties subscale of the Me and My Feelings measure^35^ was used (sample item: ‘I worry a lot’). Response options were: Never, Sometimes, and Always. Internal consistency was excellent (T1 $\alpha$ = 0.88; T2 $\alpha$ = 0.89; T3 $\alpha$ = 0.90). To create a latent variable, an item parcelling strategy was used, forming four parcels from the 10 items to address potential correlated residuals. Details on the parcelling strategy, justification, and preliminary analyses are provided in Supplementary Appendix B.

#### Socio-demographic information

The following socio-demographic data were obtained from the linked administrative data or school records: gender (0 = boys, 1 = girls); ethnicity (1 = White British, 0 = UK minoritized ethnic group); free school meal eligibility (FSM; 0 = not eligible, 1 = eligible); special educational needs (SEN; 0 = no SEN, 1 = identified as having SEN); and age in months.

### Analyses

During preliminary analysis, data were screened for missing values, skewness, and kurtosis.^36^ The Full Information Maximum Likelihood (FIML) method was used to address missing data, with variables identified as significant predictors of missingness used as auxiliary variables. Skewness and kurtosis values were calculated for each item to assess the normality of the distributions. The Maximum Likelihood estimator with Robust standard errors was implemented when absolute univariate skewness and kurtosis exceeded 2.0 and 7.0, respectively.^37^

**Figure 1:**
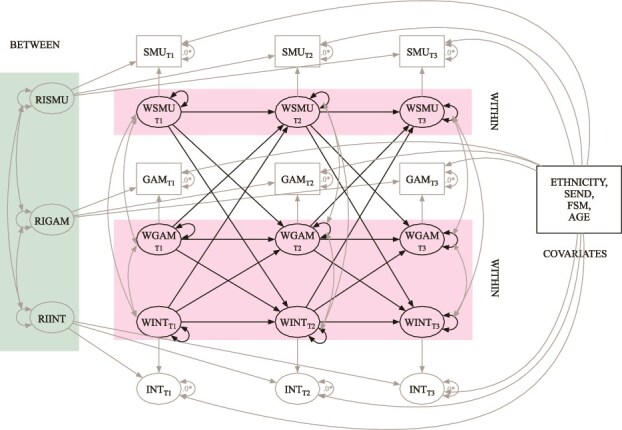
RI-CLPM model specification. SMU, social media use; GAM, gaming frequency; INT, internalizing symptoms. RI and W prefixes represent random intercepts and within components, respectively. SEND, special educational needs; FSM, free school meal eligibility; AGE, age in months at Time 1.

The main analysis employed a multigroup RI-CLPM across three measurement waves (see specification in Fig. 1). This model examined the autoregressive and cross-lagged associations between social media use, gaming frequency, and internalizing symptoms. Between-person effects were captured through covariance between random intercepts, and within-person effects through longitudinal lagged regressions and covariance between (residuals of) within-components. The analysis controlled for the potential influence of the following time-invariant sociodemographic variables as predictors of the three constructs at each wave, with these predictive effects constrained to be equal across time: ethnicity, FSM, SEN, and age at the first survey.

To ‘contextualize’ within-person cross-lagged path standardized coefficient values, 0.03, 0.07, and 0.12 were used as empirical benchmarks for small (25th percentile), moderate (50th percentile), and large (75th percentile) effects, derived from a recent meta-analysis of RI-CLPM and CLPM in psychology.^38^ Effect size ‘interpretation’ was informed by the lower-bound estimate of 0.10 proposed by Ferguson and Heene,^39^ who argue that anything below this is likely not meaningful, given that variables with little theoretical relevance to one another will correlate at or around this value in large studies.

Model fit criteria included Tucker–Lewis index (TLI) and comparative fit index (CFI) values above .95, root mean square error of approximation (RMSEA) values below .08, and standardized root mean squared residual (SRMR) values below .10.^40^ Nested model comparisons employed chi-square tests, with significant Δχ^2^ statistics (*p* < .05) indicating significant changes in model fit.

The model followed three sequential steps. First, longitudinal confirmatory factor analysis (CFA) was used to establish measurement invariance and the final measurement model. Comparisons between configural, metric, and scalar invariance measurement models utilized changes in model fit indices (ΔCFI and ΔRMSEA). The measurement model with more constraints (parsimony model) was selected when ΔCFI and ΔRMSEA fell below 0.01 and 0.015, respectively.^41^^,^^42^ Second, two models were compared: one with freely estimated parameters (Model 1) and another with within-effects parameters constrained equal across gender groups (Model 2). A chi-square difference test was used to determine the necessity of a multigroup RI-CLPM. Finally, the multigroup model (Model 1) was compared with a simpler model (Model 3), constraining similarly sized congeneric within-person paths (difference < 0.03, the empirical benchmarks for small effects in RI-CLPM)^38^ to be equal over time. A chi-square difference test evaluated whether this time-invariant constraint significantly affected model fit and determined the final model. All analyses were conducted in R 4.3 using the ‘semTools’^43^ and the ‘lavaan’^44^ packages.

## Results

Initial data screening revealed acceptable skewness and kurtosis (Supplementary Table S2). Missingness analyses supported the missing at random assumption and therefore justified the use of FIML (Supplementary Table S3). Measurement testing established longitudinal scalar invariance and multigroup metric invariance for the internalizing symptoms measure (Supplementary Tables S4 and S5).

**Figure 2:**
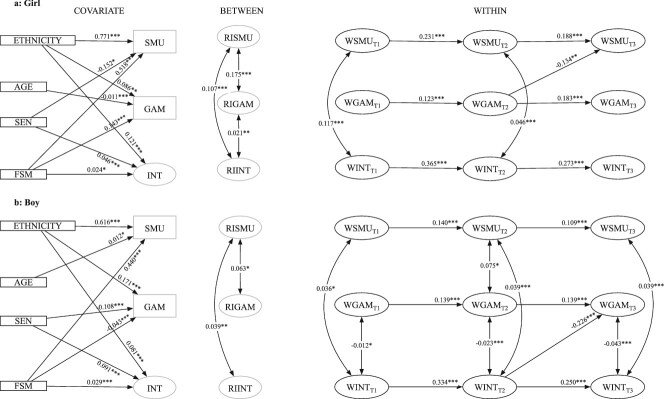
RI-CLPM for social media use, gaming frequency, and internalizing symptoms. This diagram presents the significant unstandardized paths (autoregressive, cross-lagged, and concurrent) from the multigroup RI-CLPM. The left panels depict the effects of covariate on those constructs. The middle panels show relationships between random intercepts. The right panels show within-person autoregressive, cross-lagged paths, and concurrent relationships. Nonsignificant paths were estimated but are not shown for clarity. The model was estimated as a partially stationary process; thus, congeneric paths with similar, nonsignificantly different effect sizes were constrained to be equal. SMU, social media use; GAM, gaming frequency; INT, internalizing symptoms. RI and W prefixes represent random intercepts and within components, respectively. SEND, special educational needs; FSM, free school meal eligibility. AGE = Age in months at Time 1. ^*^p < .05. ^**^p < .01. ^***^p < .001.

Comparison of Models 1 and 2 revealed that constraining parameters to be equal across gender groups significantly reduced model fit ($\Delta{\chi}^2$ = 72.226, $\Delta df$= 27, *p* < .001). Consequently, all subsequent RI-CLPM analyses are presented separately for boys and girls. Nested model comparison of the baseline multigroup RI-CLPM model with freely estimated parameters (Model 1) with the more parsimonious one (Model 3) revealed that the constraints in the latter did not significantly reduce model fit ($\Delta{\chi}^2$ = 3.36, $\Delta df$= 12, *p* = .99), and so it was selected. This final multigroup RI-CLPM model (Model 3) showed excellent fit to the data (Supplementary Table S8). The complete results are presented in Fig. 2 and Table 2.

**Table 2:** Estimates from the RI-CLPM: Cross-lagged effects, autoregressive effects, correlations between random intercepts and within-person components, and effects of covariates on latent constructs.

*Time*	*Path*	*b [95% CI]*	*p*	*Beta*	*b [95% CI]*	*p*	*Beta*
		Girl			Boy		
		Cross-lagged effects					
T1 → T2	GAM → INT	0.007 [−0.010, 0.023]	.415	0.02	0.001 [−0.024, 0.026]	.928	0.002
T2 → T3	GAM → INT	0.007 [−0.010, 0.023]	.415	0.021	−0.015 [−0.044, 0.014]	.311	−0.029
T1 → T2	GAM → SMU	−0.049 [−0.156, 0.058]	.371	−0.027	0.035 [−0.094, 0.165]	.591	0.011
T2 → T3	GAM → SMU	−0.134 [−0.218, −0.050]^**^	.002	−0.082	0.035 [−0.094, 0.165]	.591	0.013
T1 → T2	INT → GAM	0.046 [−0.116, 0.209]	.578	0.014	−0.112 [−0.285, 0.060]	.201	−0.047
T2 → T3	INT → GAM	0.046 [−0.116, 0.209]	.578	0.015	−0.226 [−0.380, −0.072]^**^	.004	−0.100
T1 → T2	INT → SMU	0.027 [−0.232, 0.286]	.839	0.005	0.142 [−0.205, 0.489]	.422	0.021
T2 → T3	INT → SMU	0.027 [−0.232, 0.286]	.839	0.005	0.142 [−0.205, 0.489]	.422	0.025
T1 → T2	SMU → GAM	−0.020 [−0.049, 0.008]	.161	−0.039	−0.010 [−0.025, 0.006]	.214	−0.029
T2 → T3	SMU → GAM	−0.020 [−0.049, 0.008]	.161	−0.035	−0.010 [−0.025, 0.006]	.214	−0.025
T1 → T2	SMU → INT	−0.001 [−0.009, 0.006]	.778	−0.006	−0.005 [−0.012, 0.002]	.152	−0.034
T2 → T3	SMU → INT	−0.001 [−0.009, 0.006]	.778	−0.006	−0.000 [−0.010, 0.010]	.960	−0.001
		Autoregression effects					
T1 → T2	GAM → GAM	0.123 [0.058, 0.188]^***^	<.001	0.117	0.139 [0.061, 0.218]^***^	<.001	0.124
T2 → T3	GAM → GAM	0.183 [0.126, 0.240]^***^	<.001	0.181	0.139 [0.061, 0.218]^***^	<.001	0.130
T1 → T2	INT → INT	0.365 [0.280, 0.449]^***^	<.001	0.351	0.334 [0.228, 0.440]^***^	<.001	0.293
T2 → T3	INT → INT	0.273 [0.185, 0.360]^***^	<.001	0.267	0.250 [0.155, 0.344]^***^	<.001	0.228
T1 → T2	SMU → SMU	0.231 [0.171, 0.291]^***^	<.001	0.255	0.140 [0.092, 0.189]^***^	<.001	0.153
T2 → T3	SMU → SMU	0.188 [0.105, 0.270]^***^	<.001	0.197	0.109 [0.048, 0.171]^***^	<.001	0.114
		Concurrent effects					
RI – RI	GAM ~ SMU	0.175 [0.081, 0.268]^***^	<.001	0.155	0.063 [0.011, 0.115]^*^	.018	0.148
T1 – T1	GAM ~ SMU	0.020 [−0.070, 0.110]	.663	0.011	0.041 [−0.012, 0.095]	.13	0.035
T2 – T2	GAM ~ SMU	−0.028 [−0.095, 0.039]	.408	−0.017	0.075 [.017, 0.132]^*^	0.01	0.063
T3 – T3	GAM ~ SMU	−0.028 [−0.095, 0.039]	.408	−0.018	0.038 [−0.013, 0.088]	.143	0.031
RI – RI	INT ~ GAM	0.021 [0.006, 0.037]^**^	.008	0.102	−0.004 [−0.014, 0.006]	.422	−0.061
T1 – T1	INT ~ GAM	−0.003 [−0.019, 0.014]	.763	−0.009	−0.012 [−0.024, −0.001]^*^	.037	−0.076
T2 – T2	INT ~ GAM	0.002 [−0.009, 0.014]	.695	0.008	−0.023 [−0.035, −0.012]^***^	<.001	−0.120
T3 – T3	INT ~ GAM	0.002 [−0.009, 0.014]	.695	0.007	−0.043 [−0.056, −0.030]^***^	<.001	−0.184
RI – RI	INT ~ SMU	0.107 [0.076, 0.138]^***^	<.001	0.265	0.039 [0.009, 0.069]^**^	.01	0.119
T1 – T1	INT ~ SMU	0.117 [0.085, 0.150]^***^	<.001	0.198	0.036 [0.003, 0.068]^*^	.031	0.065
T2 – T2	INT ~ SMU	0.046 [0.020, 0.072]^***^	<.001	0.091	0.039 [0.018, 0.060]^***^	<.001	0.072
T3 – T3	INT ~ SMU	0.020 [−0.002, 0.043]	.074	0.040	0.039 [0.018, 0.060]^***^	<.001	0.066
		Effects of covariates on latent constructs					
T1 – T3	AGE → GAM	−0.011 [−0.016, −0.006]^***^	<.001	−0.011	−0.001 [−0.004, 0.002]	.498	−0.001
T1 – T3	AGE → INT	−0.000 [−0.003, 0.002]	.673	−0.001	−0.000 [−0.002, 0.002]	.895	0.000
T1 – T3	AGE → SMU	0.009 [−0.003, 0.020]	.137	0.009	0.012 [0.002, 0.022]^*^	.015	0.012
T1 – T3	Ethnicity → GAM	0.086 [0.033, 0.139]^**^	.002	0.086	0.171 [0.141, 0.200]^***^	<.001	0.171
T1 – T3	Ethnicity → INT	0.121 [0.100, 0.141]^***^	<.001	0.288	0.081 [0.063, 0.100]^***^	<.001	0.224
T1 – T3	Ethnicity → SMU	0.771 [0.641, 0.901]^***^	<.001	0.771	0.616 [0.501, 0.730]^***^	<.001	0.616
T1 – T3	FSM → GAM	0.143 [0.101, 0.186]^***^	<.001	0.143	−0.043 [−0.069, −0.018]^***^	<.001	−0.043
T1 – T3	FSM → INT	0.024 [0.006, 0.043]^*^	.011	0.058	0.029 [0.013, 0.045]^***^	<.001	0.080
T1 – T3	FSM → SMU	0.518 [0.404, 0.631]^***^	<.001	0.518	0.440 [0.344, 0.536]^***^	<.001	0.440
T1 – T3	SEN → GAM	0.066 [−0.000, 0.132]	.051	0.066	−0.108 [−0.136, −0.080]^***^	<.001	−0.108
T1 – T3	SEN → INT	0.046 [0.025, 0.068]^***^	<.001	0.110	0.091 [0.071, 0.110]^***^	<.001	0.250
T1 – T3	SEN → SMU	−0.152 [−0.298, −0.006]^*^	.041	−0.152	−0.083 [−0.203, 0.036]	.171	−0.083

*Note*. SMU, social media use; GAM, gaming frequency; INT, internalizing symptoms; T1, 2, 3, Time 1, 2, 3; RI, random intercept; SEN, special education needs; FSM, free school meal eligibility; AGE, age in months at T1. ^*^p < .05. ^**^p < .01. ^***^p < .001.

Significant stability was observed for all within-person constructs, with scores at each timepoint predicted by scores at the preceding timepoint (T1 → T2, T2 → T3). Among these temporal relationships, internalizing symptoms demonstrated the strongest autoregressive effects, generally followed by social media use and gaming frequency. These patterns of stability were evident for both boys and girls.

Regarding social media use and internalizing symptoms, random intercepts (between-person effects) exhibited small and statistically significant positive correlations for both boys and girls. This finding was mirrored by statistically significant positive concurrent correlations at the within-person level. However, analysis of within-person cross-lagged effects demonstrated that social media use did not predict later internalizing symptoms (or vice versa) in either girls or boys.

Regarding social media use and gaming frequency, the random intercepts exhibited small, statistically significant positive correlations for both boys and girls. At the within-person level, concurrent correlations were predominantly nonsignificant, with the sole exception of a statistically significant but very small association at T2 for boys. Analysis of within-person cross-lagged effects revealed that social media use did not predict subsequent gaming frequency for either boys or girls. However, gaming frequency at T2 negatively predicted social media use at T3 for girls, with a moderate effect size that exceeded the empirical benchmark of 0.07.

Finally, regarding internalizing symptoms and gaming frequency, the random intercepts exhibited small, statistically significant positive correlations for girls but not for boys. At the within-person level, concurrent correlations were nonsignificant for girls, while boys showed statistically significant negative correlations of small magnitude. Analysis of within-person cross-lagged effects revealed that gaming frequency did not significantly predict subsequent internalizing symptoms for boys or girls. However, internalizing symptoms at T2 inversely predicted gaming frequency at T3 for boys, with a moderate effect size that met the lower-bound estimate of 0.10, meaning the effect was likely meaningful.

Following the above, sensitivity analyses were undertaken to assess the robustness of these findings to the distinction between active and passive social media use (i.e. replacement of overall time spent on social media in the above models with time spent engaging in active or passive activities, respectively). These analyses replicated the above findings, with the following exceptions: for boys, passive social media use at T1 inversely predicted internalizing symptoms at T2 (moderate effect), and gaming frequency positively predicted later passive social media use (small effect); for girls, the negative association between gaming and subsequent social media use reduced in both the passive and active models, and a new negative association between active social media use and later gaming frequency (small effect) was found (Supplementary Tables S11 and S12).

## Discussion

### Main findings of this study

Longitudinal relationships varied by gender, such that more frequent gaming at T2 predicted less time spent on social media use at T3 in girls (but not boys) and more frequent internalizing symptoms at T2 predicted reductions in gaming frequency at T3 in boys (but not girls). There was no evidence that time spent on social media or gaming frequency predicted later internalizing symptoms among girls or boys. Sensitivity analyses that distinguished active versus passive social media use replicated these findings.

### What is already known on this topic

There is a widespread view that digital technology use (e.g. social media use, gaming) is the driving causal factor in adolescent MHDs.^5^ Research findings are, however, very mixed,^8–10^^,^^16^^,^^17^ with firm conclusions precluded by a range of methodological and analytic limitations.

### What this study adds

This study adds considerable methodological and analytical rigour, including the use of a longitudinal design, a very large and heterogeneous sample, control for confounding factors, inclusion of sensitivity analyses that distinguish active versus passive social media use, and application of the RI-CLPM to enable separation of between-person from within-person effects. These features enable greater clarity and precision vis-à-vis the presence, predominance, and sign of causal relationships between adolescent technology use and mental health.

The lack of evidence linking social media use or gaming frequency to later internalizing symptoms suggests that these activities may not play a causal role in the development of adolescent mental health difficulties. Our findings challenge the widespread assumption that time spent on these technologies is inherently harmful and highlight the need for more nuanced perspectives that consider the context and individual differences in their use. However, distinguishing between active and passive use of social media played a limited role in our overall findings. While this is in contrast to some research,^24^^,^^45^ the results support the notion that the distinction may be overly broad and does not sufficiently predict mental health.^46^ At the same time, our findings did not support the idea that internalizing symptoms predict later social media use.

A notable observation in the study was that higher levels of internalizing symptoms among boys reported reduced gaming frequency 1 year later. A common symptom of depression, including in children, is withdrawal from previously enjoyed activities,^47^ which could explain this finding. Alternatively, while parents’ attitudes towards gaming were not directly measured in this study, another hypothesis, supported by existing literature,^48–50^ is that parents of these young people may have restricted their gaming time due to concerns about its contribution to internalizing symptoms or the loss of other social activities. For instance, research indicates that parents with more negative attitudes towards video games tend to be more controlling when regulating their child’s gaming^49^ and that parents are more likely to set stricter gaming rules for boys than for girls.^50^ Investigating the role of parental restrictions in this dynamic presents an important avenue for future research.

### Limitations of this study

This study relied on self-reported data and a 12-month lag between measurements, which may limit the detection of important shorter-term, reciprocal associations between digital technology use and internalizing symptoms. Adolescent engagement with social media and gaming, as well as their emotional states, is known to exhibit considerable variability on a daily, or even hourly, basis.^51^^,^^52^ Consequently, experiences such as the immediate emotional uplift from positive social media feedback, the distress following a negative online interaction, or the temporary mood regulation achieved via a gaming session are by their nature transient, unfolding over minutes or hours, not months. To capture shorter-term impacts and improve ecological validity, future research should employ ecological momentary assessment designs. Interestingly, a recent study utilizing such methods found no significant effects of time spent on social media on suicidal ideation but did report significant (albeit small and not always consistent) within-person effects of negative and positive social media experiences (i.e. on days characterized by more frequent negative social media experiences than usual, young people were more likely to report suicidal ideation; however, more positive social media experiences than usual were associated with a lower likelihood of suicidal ideation).^53^ The annual nature of our data inherently limits our capacity to capture the granularity of these more immediate and potentially bi-directional associations. Future research employing such intensive longitudinal designs is therefore crucial to disentangle these fine-grained, short-term dynamics from the more stable, longer-term associations that studies like ours are better suited to identify.

Our study also did not differentiate between various types of games or social media; this is important because certain games are more strongly associated with problematic gaming.^54^ Online gaming is often included within the broader definition of social media,^55^ and platforms like Discord or Twitch are integral to the gaming community. The lack of differentiation between these platforms may obscure important findings and could partly explain why more frequent gaming among girls predicted decreases in later social media use. In addition, there are contextual factors that likely moderate the psychological effects of video gaming—such as social context (e.g. solo vs. multiplayer), player motivation (e.g. escapism, competition), and timing of play,^56^ which were not captured in the current study. Similarly, different social media platforms vary in features and user engagement patterns, which may influence their impact on mental health in distinct ways. Additionally, the reliance on time-based measures for social media and gaming overlooks other important dimensions, such as the purpose of use, emotional responses, or the specific nature of interactions. Similarly, while some evidence suggests that the distinction between ‘active’ and ‘passive’ social media use may offer more nuanced insights than total time spent,^24^ it remains narrow in scope.^46^ Finally, while the large and diverse sample enhances generalizability, it is still limited to the UK context, and findings may not fully apply to other cultural settings.

## Supplementary Material

Supplementary_Materials_fdaf150

## Data Availability

The #BeeWell survey data will be released publicly in an anonymized format in 2026. This release date is necessary to comply with participants’ data withdrawal rights, which extend until that time. Maintaining a secure, pseudonymized version of the data is required during this period. Administrative data linked to the survey, including sex and free school meal eligibility, will remain permanently confidential and will not be released, in accordance with data sharing agreements established with participating Local Authorities. For inquiries regarding access to the #BeeWell data, please contact Professor Neil Humphrey at neil.humphrey@manchester.ac.uk.

## Notes

**Qiqi Cheng**, Researcher

**Margarita Panayiotou**, Researcher

**Turi Reiten Finserås**, Researcher

**Amanda Iselin Olesen Andersen**, Researcher

**Neil Humphrey**, Researcher
