# Supplementary material for: How do social media use, gaming frequency, and internalizing symptoms predict each other over time in early-to-middle adolescence?
Source: J Public Health (Oxf). 2025 Dec 5;48(1):59–69. doi: 10.1093/pubmed/fdaf150 (PMC13017292; doi:10.1093/pubmed/fdaf150)
Supplement: Supplementary_Materials_fdaf150 [file supplementary_materials_fdaf150.docx]

**How Do Social Media Use, Gaming Frequency, and Internalising Symptoms Influence Each Other Over Time in Early-to-Middle Adolescence?: Supplementary Materials**

Supplementary materials include the following appendices: Appendix A) Data Screening; B) Parcelling and Measurement Invariance; C) Baseline RI-CLPM; D) Model Fit; and E) Sensitivity Analysis.

# A): Data Screening

To handle missing data, Full Information Maximum Likelihood (FIML) was utilised. FIML is a robust approach appropriate when data are missing at random (MAR). To confirm if the data is MAR, Little's MCAR test was conducted. While the overall chi-square was statistically significant (χ² = 23618.91, df = 20969, p < .001), the normed chi-square (χ²/df = 1.13) was low, suggesting that the data deviated only slightly from being missing completely at random (MCAR). Logistic regression analyses were then performed to identify predictors of missingness, with results (detailed in Supplementary Table S3) indicating that gender, special educational needs, free school meal eligibility, and ethnicity were significant predictors. The identification of these systematic predictors of missingness provides support for the MAR assumption, as missingness appears to be related to observed characteristics. Consequently, FIML was deemed an appropriate method, as it leverages all available data and incorporates these identified predictors as auxiliary variables, thereby mitigating potential bias and yielding more efficient estimates compared to traditional deletion methods under the MAR assumption(Enders, 2022).

**Table S1**

*Participant Characteristics*

| Characteristic | Percentage/Mean | Number of Observations/SD |
| --- | --- | --- |
| Age at Time 1 | 12 years 7 months | 3.58 months |
|  |  |  |
| Gender |  |  |
| Girls | 51% | 12975 |
| Boys | 49% | 12654 |
|  |  |  |
| SEN |  |  |
| Yes | 17% | 4265 |
| No | 83% | 21364 |
|  |  |  |
| FSM |  |  |
| Yes | 29% | 7337 |
| No | 71% | 18292 |
|  |  |  |
| Ethnicity |  |  |
| White British | 66% | 16825 |
| Minority | 34% | 8804 |

*Note.* SEN = special educational needs. FSM = free school meal eligibility.

**Table S2**

*Missing Data, Skewness, and Kurtosis of Indicators*

|  | Missingness | |  |  |
| --- | --- | --- | --- | --- |
| Variable | N | % | Skewness | Kurtosis |
| SMU_T1 | 7482 | 32.0 | 0.03 | 1.88 |
| INT1_T1 | 7222 | 30.8 | 0.25 | 2.34 |
| INT2_T1 | 7284 | 31.1 | -0.09 | 2.84 |
| INT3_T1 | 7384 | 31.5 | 0.86 | 2.72 |
| INT4_T1 | 7325 | 31.3 | 0.64 | 2.25 |
| INT5_T1 | 7276 | 31.1 | 0.46 | 2.09 |
| INT6_T1 | 7310 | 31.2 | 0.34 | 1.84 |
| INT7_T1 | 7277 | 31.1 | 0.48 | 1.82 |
| INT8_T1 | 7299 | 31.2 | 0.31 | 1.92 |
| INT9_T1 | 7300 | 31.2 | 0.50 | 2.15 |
| INT10_T1 | 7344 | 31.4 | 0.85 | 2.71 |
| GAM_T1 | 7682 | 32.8 | -1.49 | 3.57 |
| SMU_T2 | 8865 | 37.9 | -0.07 | 1.97 |
| INT1_T2 | 8811 | 37.6 | 0.36 | 2.33 |
| INT2_T2 | 8867 | 37.9 | 0.02 | 2.67 |
| INT3_T2 | 8932 | 38.2 | 0.90 | 2.76 |
| INT4_T2 | 8898 | 38.0 | 0.73 | 2.28 |
| INT5_T2 | 8849 | 37.8 | 0.47 | 2.02 |
| INT6_T2 | 8884 | 37.9 | 0.36 | 1.80 |
| INT7_T2 | 8845 | 37.8 | 0.50 | 1.81 |
| INT8_T2 | 8858 | 37.8 | 0.39 | 1.90 |
| INT9_T2 | 8871 | 37.9 | 0.55 | 2.17 |
| INT10_T2 | 8890 | 38.0 | 0.95 | 2.85 |
| GAM_T2 | 9010 | 38.5 | -1.19 | 2.75 |
| SMU_T3 | 9594 | 41.0 | -0.06 | 2.01 |
| INT1_T3 | 9567 | 40.9 | 0.48 | 2.35 |
| INT2_T3 | 9618 | 41.1 | 0.13 | 2.46 |
| INT3_T3 | 9667 | 41.3 | 1.09 | 3.13 |
| INT4_T3 | 9636 | 41.2 | 0.84 | 2.43 |
| INT5_T3 | 9616 | 41.1 | 0.55 | 2.12 |
| INT6_T3 | 9645 | 41.2 | 0.43 | 1.89 |
| INT7_T3 | 9601 | 41.0 | 0.52 | 1.86 |
| INT8_T3 | 9620 | 41.1 | 0.48 | 1.95 |
| INT9_T3 | 9638 | 41.2 | 0.58 | 2.23 |
| INT10_T3 | 9645 | 41.2 | 1.07 | 3.09 |
| GAM_T3 | 9764 | 41.7 | -1.04 | 2.40 |

*Note.* SMU = Social media usage. GAM = Gaming Frequency. INT = Internalising symptoms. T1, 2, 3 = Time 1, 2, 3.

**Table S3**

*Odds Ratios Pertaining to Missingness for Covariates for Social Media Use, Internalising Symptoms and Gaming Frequency.*

| Variable | Female | Ethnicity | SEN | FSM | AGE |
| --- | --- | --- | --- | --- | --- |
| SMU_T1 | 0.86 ** | 0.56 *** | 1.45 *** | 1.2 ** | 0.99 |
| INT1_T1 | 0.75 *** | 0.56 *** | 1.74 *** | 1.14 * | 1 |
| INT2_T1 | 0.76 *** | 0.58 *** | 1.81 *** | 1.15 * | 1 |
| INT3_T1 | 0.82 *** | 0.57 *** | 1.82 *** | 1.18 ** | 1 |
| INT4_T1 | 0.78 *** | 0.58 *** | 1.75 *** | 1.13 * | 0.99 |
| INT5_T1 | 0.77 *** | 0.57 *** | 1.74 *** | 1.14 * | 0.99 |
| INT6_T1 | 0.8 *** | 0.58 *** | 1.74 *** | 1.14 * | 0.99 |
| INT7_T1 | 0.79 *** | 0.58 *** | 1.8 *** | 1.16 * | 0.99 |
| INT8_T1 | 0.78 *** | 0.57 *** | 1.75 *** | 1.14 * | 0.99 |
| INT9_T1 | 0.78 *** | 0.56 *** | 1.85 *** | 1.14 * | 0.99 |
| INT10_T1 | 0.77 *** | 0.57 *** | 1.73 *** | 1.15 * | 0.99 |
| GAM_T1 | 0.9 * | 0.58 *** | 1.55 *** | 1.23 *** | 1 |
| SMU_T2 | 0.85 ** | 0.73 *** | 1.5 *** | 1.37 *** | 1.02 * |
| INT1_T2 | 0.87 * | 0.79 *** | 1.51 *** | 1.31 *** | 1.01 |
| INT2_T2 | 0.9 | 0.77 *** | 1.56 *** | 1.29 *** | 1.01 |
| INT3_T2 | 0.97 | 0.76 *** | 1.58 *** | 1.28 *** | 1.01 |
| INT4_T2 | 0.92 | 0.77 *** | 1.56 *** | 1.24 *** | 1.01 |
| INT5_T2 | 0.89 | 0.76 *** | 1.57 *** | 1.29 *** | 1.01 |
| INT6_T2 | 0.89 * | 0.77 *** | 1.52 *** | 1.27 *** | 1.01 |
| INT7_T2 | 0.88 * | 0.75 *** | 1.58 *** | 1.29 *** | 1.01 |
| INT8_T2 | 0.9 | 0.76 *** | 1.53 *** | 1.3 *** | 1.01 |
| INT9_T2 | 0.89 * | 0.76 *** | 1.53 *** | 1.3 *** | 1.01 |
| INT10_T2 | 0.89 * | 0.76 *** | 1.55 *** | 1.31 *** | 1.01 |
| GAM_T2 | 0.86 ** | 0.74 *** | 1.52 *** | 1.37 *** | 1.01 |
| SMU_T3 | 0.82 *** | 0.77 *** | 1.54 *** | 1.21 ** | 1 |
| INT1_T3 | 0.87 * | 0.76 *** | 1.45 *** | 1.27 *** | 1.01 |
| INT2_T3 | 0.89 * | 0.74 *** | 1.47 *** | 1.28 *** | 1.01 |
| INT3_T3 | 0.91 | 0.74 *** | 1.51 *** | 1.27 *** | 1.01 |
| INT4_T3 | 0.86 ** | 0.72 *** | 1.48 *** | 1.26 *** | 1.01 |
| INT5_T3 | 0.86 ** | 0.74 *** | 1.49 *** | 1.25 *** | 1.01 |
| INT6_T3 | 0.87 * | 0.74 *** | 1.52 *** | 1.23 *** | 1.01 |
| INT7_T3 | 0.84 ** | 0.76 *** | 1.44 *** | 1.25 *** | 1.01 |
| INT8_T3 | 0.87 * | 0.75 *** | 1.49 *** | 1.24 *** | 1.01 |
| INT9_T3 | 0.87 * | 0.74 *** | 1.51 *** | 1.24 *** | 1.01 |
| INT10_T3 | 0.85 ** | 0.74 *** | 1.52 *** | 1.25 *** | 1.01 |
| GAM_T3 | 0.85 ** | 0.75 *** | 1.58 *** | 1.22 *** | 1 |

*Note.* SMU = Social media usage. GAM = Gaming Frequency. INT = Internalising symptoms. T1, 2, 3 = Time 1, 2, 3.

* p < .05, ** p < .01, *** p < .001

# B): Parcelling and Measurement Invariance

The 10-item emotional difficulties subscale of the "Me and My Feelings" measure (sample item: "I worry a lot," three-point response scale: "Never," "Sometimes," "Always") was used to assess internalising symptoms (Deighton et al., 2013). A longitudinal confirmatory factor analysis (CFA) of the 10 items showed acceptable overall fit (CFI = 0.928, TLI = 0.916, RMSEA = 0.056, SRMR = 0.033), but dynamic fit indices (McNeish & Wolf, 2023) and modification indices suggested potential correlated residuals, which can bias parameter estimates. Exploratory graph analysis (EGA) confirmed the unidimensionality of the construct, consistent with prior psychometric study using wave one data from the same project (Black et al., 2024). To address the potential correlated residuals, we employed item parcelling (Little et al., 2022, Hall et al., 1999), a method for mitigating the influence of potential correlated residuals. Items were grouped into four parcels based primarily on the pattern of modification indices to minimize these correlated residuals: Parcel 1 (items 1-3), Parcel 2 (items 4-6), Parcel 3 (items 7-8), and Parcel 4 (items 9-10). After parcelling, the longitudinal CFA suggests good overall fit (e.g., the configural invariance longitudinal CFI = 0.997, TLI = 0.994, RMSEA = 0.024, SRMR = 0.011, see table S4 below). The measurement invariance tests across three waves were conducted. Results indicate the longitudinal scalar and group metric invariance were supported (see Table S4 and S5 below ).

**Table S4.**

*Longitudinal Measurement Invariance of* *Internalising Symptoms Across Three Waves*

| Model tested | $\chi^{2}$ | $df$ | $p$ | ${\Delta\chi}^{2}$ | $\Delta df$ | $p$ | RMSEA | RMSEA 90% CI | CFI | $\Delta$CFI | TLI/ NNFI | $\Delta$TLI | SRMR | Pass? |
| --- | --- | --- | --- | --- | --- | --- | --- | --- | --- | --- | --- | --- | --- | --- |
| Null model | 92457.222 | 66 | <.001 |  |  |  |  |  |  |  |  |  |  |  |
|  |  |  |  | Measurement model | | | |  |  |  |  |  |  |  |
| Configural invariance | 278.040 | 39 | <.001 |  |  |  | .024 | (.021, .027) | .997 |  | .994 |  | .011 | Yes |
| Metric invariance | 322.244 | 45 | <.001 | 44.402 | 6 | <.001 | .024 | (.021, .027) | .996 | -0.001 | .995 | 0.001 | .012 | Yes |
| Scalar invariance | 369.960 | 51 | <.001 | 47.778 | 6 | <.001 | .024 | (.021, .026) | .996 | 0 | .995 | 0 | .013 | Yes |

*Note.* $\chi^{2}$ = chi-square; df = degrees of freedom; RMSEA = root mean square error of approximation; CFI = Comparative Fit Index; TLI = Tucker-Lewis Index; SRMR = Standardized Root Mean Squared Residual.

**Table S5.**

*Omnibus Measurement Invariance of Internalising Symptoms Across Gender Groups and Time Points*

| Model tested | $\chi^{2}$ | $df$ | $p$ | ${\Delta\chi}^{2}$ | $\Delta df$ | $p$ | RMSEA | RMSEA 90% CI | CFI | $\Delta$CFI | TLI/ NNFI | $\Delta$TLI | SRMR | Pass? |
| --- | --- | --- | --- | --- | --- | --- | --- | --- | --- | --- | --- | --- | --- | --- |
| Null model | 76235.423 | 132 | <.001 |  |  |  |  |  |  |  |  |  |  |  |
|  |  |  |  | Measurement model | | | |  |  |  |  |  |  |  |
| Configural invariance | 323.800 | 78 | <.001 |  |  |  | .023 | (.020, .027) | .996 |  | .994 |  | .013 |  |
| Metric invariance | 660.538 | 93 | <.001 | 393.93 | 15 | < .001 | .030 | (.027, .033) | .993 | -0.003 | .990 | -0.004 | .022 | Yes |
| Longitudinal and group Scalar invariance | 2837.791 | 108 | <.001 | 3946 | 15 | < .001 | .056 | (.053, .058) | .972 | -0.021 | .965 | -0.025 | .035 | No |
| Longitudinal scalar and group metric invariance | 762.183 | 105 | <.001 | 101.65 | 36 | < .001 | .030 | (.028, .033) | .992 | -0.001 | .990 | 0 | .023 | Yes |

*Note.* $\chi^{2}$ = chi-square; df = degrees of freedom RMSEA = root mean square error of approximation; CFI = Comparative Fit Index; TLI = Tucker-Lewis Index; SRMR = Standardized Root Mean Squared Residual.

# C): Baseline Single Group Analysis

In this appendix, single-group RI-CLPM models (i.e., not separated by gender) were also estimated. Analysis of the within-person cross-lagged effects revealed no statistically significant predictive relationships between social media use and subsequent internalizing symptoms, nor between gaming frequency and subsequent internalizing symptoms, for the sample as a whole. Reciprocally, internalizing symptoms did not significantly predict later changes in either social media use or gaming frequency.

**Table S6.**

*Latent Variance–Covariance Matrix and Latent Means*

| Construct | SMU_T1 | SMU_T2 | SMU_T3 | GAM_T1 | GAM_T2 | GAM_T3 | INT_T1 | INT_T2 | INT_T3 |
| --- | --- | --- | --- | --- | --- | --- | --- | --- | --- |
| SMU_T1 | 1.00 |  |  |  |  |  |  |  |  |
| SMU_T2 | 0.532*** | 1.00 |  |  |  |  |  |  |  |
| SMU_T3 | 0.443*** | 0.532*** | 1.00 |  |  |  |  |  |  |
| GAM_T1 | 0.007 | -0.016 | -0.006 | 1.00 |  |  |  |  |  |
| GAM_T2 | -0.040*** | -0.026* | -0.047*** | 0.506*** | 1.00 |  |  |  |  |
| GAM_T3 | -0.038** | -0.048*** | -0.033** | 0.451*** | 0.538*** | 1.00 |  |  |  |
| INT_T1 | 0.221*** | 0.190*** | 0.166*** | -0.111*** | -0.130*** | -0.124*** | 1.00 |  |  |
| INT_T2 | 0.165*** | 0.213*** | 0.173*** | -0.108*** | -0.163*** | -0.162*** | 0.658*** | 1.00 |  |
| INT_T3 | 0.146*** | 0.166*** | 0.186*** | -0.108*** | -0.140*** | -0.170*** | 0.517*** | 0.601*** | 1.00 |
| Means | 4.34 | 4.68 | 4.73 | 3.41 | 3.27 | 3.20 | 0.67 | 0.66 | 0.63 |
| Std. Dev. | 2.51 | 2.37 | 2.31 | 1.06 | 1.13 | 1.17 | 0.42 | 0.44 | 0.45 |

*Note.* Results are based on the effects-coded method of identification with the scalar invariant across time. SMU = Social media usage. GAM = Gaming Frequency. INT = Internalising symptoms. T1, 2, 3 = Time 1, 2, 3.

* p < .05, ** p < .01, *** p < .001

**Table S7.**

*Results of the Baseline Partial Stationary RI-CLPM*

| Time | Path | *b* | *p* | *CI* | *beta* |
| --- | --- | --- | --- | --- | --- |
|  |  | Cross-lagged Pathways | | | |
| T1 to T2 | GAM → INT | 0.007 | 0.335 | [-0.008, 0.023] | 0.019 |
| T2 to T3 | GAM → INT | -0.007 | 0.405 | [-0.023, 0.009] | -0.018 |
| T1 to T2 | GAM → SMU | -0.027 | 0.555 | [-0.116, 0.063] | -0.012 |
| T2 to T3 | GAM → SMU | -0.101** | 0.003 | [-0.169, -0.034] | -0.051 |
| T1 to T2 | INT → GAM | -0.094 | 0.087 | [-0.202, 0.014] | -0.033 |
| T2 to T3 | INT → GAM | -0.094 | 0.087 | [-0.202, 0.014] | -0.034 |
| T1 to T2 | INT → SMU | 0.038 | 0.713 | [-0.166, 0.242] | 0.006 |
| T2 to T3 | INT → SMU | 0.038 | 0.713 | [-0.166, 0.242] | 0.007 |
| T1 to T2 | SMU → GAM | -0.015 | 0.059 | [-0.030, 0.001] | -0.034 |
| T2 to T3 | SMU → GAM | -0.015 | 0.059 | [-0.030, 0.001] | -0.03 |
| T1 to T2 | SMU → INT | -0.003 | 0.267 | [-0.008, 0.002] | -0.019 |
| T2 to T3 | SMU → INT | -0.003 | 0.267 | [-0.008, 0.002] | -0.016 |
|  |  | Autoregressive Effects | | | |
| T1 to T2 | GAM → GAM | 0.119*** | < .001 | [0.062, 0.175] | 0.112 |
| T2 to T3 | GAM → GAM | 0.178*** | < .001 | [0.128, 0.228] | 0.172 |
| T1 to T2 | INT → INT | 0.343*** | < .001 | [0.279, 0.407] | 0.319 |
| T2 to T3 | INT → INT | 0.259*** | < .001 | [0.200, 0.318] | 0.246 |
| T1 to T2 | SMU → SMU | 0.180*** | < .001 | [0.141, 0.219] | 0.198 |
| T2 to T3 | SMU → SMU | 0.144*** | < .001 | [0.090, 0.197] | 0.15 |

*Notes.* $\chi^{2}$ = 3480.818, df = 190, RMSEA = 0.034 (.033, . 035), CFI =0 975, TLI = 0.968, SRMR = 0.028. N = 25629. SMU = Social media usage. GAM = Gaming Frequency. INT = Internalising symptoms. T1, 2, 3 = Time 1, 2, 3.

*p < .05, **p <.01, ***p <.00

# D): Model Fit

**Table S8**

*Model fits and comparisons for RI-CLPMs*

| Model | $\chi^{2}$ | $df$ | RMSEA (90% CI) | CFI | TLI | SRMR | ${\Delta\chi}^{2}$ | $\Delta df$ | $p$ |
| --- | --- | --- | --- | --- | --- | --- | --- | --- | --- |
|  | *Model fits* | | | | | | *Comparison with Model 1* | | |
| Model 1 | 2175.974 | 339 | .027(.025, .028) | .983 | .978 | .027 | - | - | - |
| Model 2 | 2228.063 | 366 | .026(.025, .027) | .983 | .979 | .028 | 72.226 | 27 | < .001 |
| Model 3 | 2164.645 | 351 | .026(.025, .027) | .983 | .979 | .027 | 3.3608 | 12 | .992 |

*Note*. Model 1 = freely estimated parameters. Model 2 = within-effects parameters constrained equal across gender groups. Model 3 = time-invariant model with similarly sized congeneric within-person paths constrained equal. Model comparisons were conducted using chi-square difference tests, with p < .05 indicating significant changes in model fit.

# E): Sensitivity Analysis

This appendix presents a series of sensitivity analyses to assess the robustness of the findings reported in the main text. We explored alternative model specifications for key variables. For Internalising Symptoms, we present results using the original 10 items with correlated residuals (Table S9) and a manifest variable of averaged scores (Table S10), as opposed to the parcelling approach used in the main analysis. For social media use, we examined models incorporating active (Table S11) and passive (Table S12) social media usage, instead of the overall time spent used in the main text. To check the sample selection bias, sensitivity analyses were conducted using samples with at least two waves (Table S13) and at least three waves of data (Table S14). Further, we included the original six-category gaming frequency variable (Table S15) instead of the four-category version used in the main manuscript. Finally, we report results using multiple imputation with 50 imputed datasets for participant with at least two survey waves(Table S16) and participants with at least one waves (Table S17), as a comparison to the FIML approach used in the main manuscript. These analyses, detailed in Tables S9-S17 below, provide further support for the robustness of the primary results.

**Table S9**

*Analysis Using Original 10 Internalising Symptoms Items with Correlated Residuals Covarying*

|  |  | Girls | | | |  | Boys | | | |
| --- | --- | --- | --- | --- | --- | --- | --- | --- | --- | --- |
| Time | Path | *b* | *p* | *CI* | *beta* |  | *b* | *p* | *CI* | *beta* |
|  |  | Cross-lagged Pathways | | | | | | | | |
| T1 to T2 | GAM → INT | 0.006 | 0.459 | [-0.011, 0.023] | 0.018 |  | 0.003 | 0.828 | [-0.022, 0.028] | 0.005 |
| T2 to T3 | GAM → INT | 0.006 | 0.459 | [-0.011, 0.023] | 0.019 |  | -0.014 | 0.341 | [-0.044, 0.015] | -0.027 |
| T1 to T2 | GAM → SMU | -0.049 | 0.371 | [-0.156, 0.058] | -0.027 |  | 0.035 | 0.596 | [-0.094, 0.164] | 0.011 |
| T2 to T3 | GAM → SMU | -0.134** | 0.002 | [-0.217, -0.050] | -0.082 |  | 0.035 | 0.596 | [-0.094, 0.164] | 0.013 |
| T1 to T2 | INT → GAM | 0.04 | 0.637 | [-0.125, 0.204] | 0.013 |  | -0.095 | 0.272 | [-0.263, 0.074] | -0.04 |
| T2 to T3 | INT → GAM | 0.04 | 0.637 | [-0.125, 0.204] | 0.013 |  | -0.214** | 0.006 | [-0.367, -0.061] | -0.095 |
| T1 to T2 | INT → SMU | 0.044 | 0.738 | [-0.216, 0.304] | 0.008 |  | 0.125 | 0.486 | [-0.227, 0.478] | 0.019 |
| T2 to T3 | INT → SMU | 0.044 | 0.738 | [-0.216, 0.304] | 0.009 |  | 0.125 | 0.486 | [-0.227, 0.478] | 0.022 |
| T1 to T2 | SMU → GAM | -0.02 | 0.166 | [-0.049, 0.008] | -0.038 |  | -0.01 | 0.207 | [-0.025, 0.005] | -0.03 |
| T2 to T3 | SMU → GAM | -0.02 | 0.166 | [-0.049, 0.008] | -0.034 |  | -0.01 | 0.207 | [-0.025, 0.005] | -0.025 |
| T1 to T2 | SMU → INT | -0.001 | 0.725 | [-0.009, 0.006] | -0.008 |  | -0.006 | 0.111 | [-0.013, 0.001] | -0.037 |
| T2 to T3 | SMU → INT | -0.001 | 0.725 | [-0.009, 0.006] | -0.007 |  | 0 | 0.962 | [-0.010, 0.010] | -0.001 |
|  |  | Autoregressive Effects | | | | | | | | |
| T1 to T2 | GAM → GAM | 0.123*** | 0 | [0.058, 0.188] | 0.116 |  | 0.140*** | 0 | [0.061, 0.218] | 0.125 |
| T2 to T3 | GAM → GAM | 0.183*** | 0 | [0.126, 0.240] | 0.181 |  | 0.140*** | 0 | [0.061, 0.218] | 0.13 |
| T1 to T2 | INT → INT | 0.385*** | 0 | [0.298, 0.473] | 0.371 |  | 0.354*** | 0 | [0.248, 0.461] | 0.311 |
| T2 to T3 | INT → INT | 0.296*** | 0 | [0.207, 0.385] | 0.293 |  | 0.256*** | 0 | [0.161, 0.351] | 0.235 |
| T1 to T2 | SMU → SMU | 0.231*** | 0 | [0.171, 0.291] | 0.255 |  | 0.140*** | 0 | [0.092, 0.189] | 0.153 |
| T2 to T3 | SMU → SMU | 0.188*** | 0 | [0.105, 0.270] | 0.197 |  | 0.109*** | 0.001 | [0.048, 0.171] | 0.114 |

*Note.* SMU = Social media usage. GAM = Gaming Frequency. INT = Internalising symptoms. T1, 2, 3 = Time 1, 2, 3.

* p < .05, ** p < .01, *** p < .001

**Table S10**

*Analysis Using the Manifest Variable (Averaged Scores for Internalising symptoms)*

|  |  | Girls | | | |  | Boys | | | |
| --- | --- | --- | --- | --- | --- | --- | --- | --- | --- | --- |
| Time | Path | *b* | *p* | *CI* | *beta* |  | *b* | *p* | *CI* | *beta* |
|  |  | Cross-lagged Pathways | | | | | | | | |
| T1 to T2 | GAM → INT | 0.007 | 0.377 | [-0.009, 0.024] | 0.019 |  | 0 | 0.99 | [-0.026, 0.026] | 0 |
| T2 to T3 | GAM → INT | 0.007 | 0.377 | [-0.009, 0.024] | 0.02 |  | -0.019 | 0.209 | [-0.049, 0.011] | -0.033 |
| T1 to T2 | GAM → SMU | -0.048 | 0.375 | [-0.155, 0.059] | -0.027 |  | 0.032 | 0.632 | [-0.099, 0.163] | 0.01 |
| T2 to T3 | GAM → SMU | -0.135** | 0.002 | [-0.218, -0.051] | -0.082 |  | 0.032 | 0.632 | [-0.099, 0.163] | 0.012 |
| T1 to T2 | INT → GAM | 0.044 | 0.464 | [-0.074, 0.162] | 0.016 |  | -0.067 | 0.312 | [-0.197, 0.063] | -0.033 |
| T2 to T3 | INT → GAM | 0.044 | 0.464 | [-0.074, 0.162] | 0.016 |  | -0.195** | 0.002 | [-0.318, -0.071] | -0.096 |
| T1 to T2 | INT → SMU | 0.018 | 0.858 | [-0.178, 0.214] | 0.004 |  | 0.115 | 0.373 | [-0.139, 0.369] | 0.02 |
| T2 to T3 | INT → SMU | 0.018 | 0.858 | [-0.178, 0.214] | 0.004 |  | 0.115 | 0.373 | [-0.139, 0.369] | 0.023 |
| T1 to T2 | SMU → GAM | -0.02 | 0.156 | [-0.049, 0.008] | -0.039 |  | -0.01 | 0.192 | [-0.026, 0.005] | -0.031 |
| T2 to T3 | SMU → GAM | -0.02 | 0.156 | [-0.049, 0.008] | -0.035 |  | -0.01 | 0.192 | [-0.026, 0.005] | -0.026 |
| T1 to T2 | SMU → INT | 0.001 | 0.864 | [-0.007, 0.008] | 0.003 |  | -0.004 | 0.248 | [-0.012, 0.003] | -0.025 |
| T2 to T3 | SMU → INT | 0.001 | 0.864 | [-0.007, 0.008] | 0.003 |  | 0.002 | 0.645 | [-0.008, 0.013] | 0.012 |
|  |  | Autoregressive Effects | | | | | | | | |
| T1 to T2 | GAM → GAM | 0.123*** | 0 | [0.058, 0.188] | 0.117 |  | 0.141*** | 0 | [0.062, 0.220] | 0.126 |
| T2 to T3 | GAM → GAM | 0.183*** | 0 | [0.126, 0.241] | 0.181 |  | 0.141*** | 0 | [0.062, 0.220] | 0.132 |
| T1 to T2 | INT → INT | 0.303*** | 0 | [0.236, 0.370] | 0.293 |  | 0.248*** | 0 | [0.170, 0.327] | 0.228 |
| T2 to T3 | INT → INT | 0.223*** | 0 | [0.155, 0.292] | 0.222 |  | 0.208*** | 0 | [0.132, 0.283] | 0.191 |
| T1 to T2 | SMU → SMU | 0.230*** | 0 | [0.171, 0.290] | 0.255 |  | 0.141*** | 0 | [0.092, 0.189] | 0.154 |
| T2 to T3 | SMU → SMU | 0.187*** | 0 | [0.105, 0.269] | 0.196 |  | 0.110*** | 0 | [0.049, 0.171] | 0.115 |

*Note.* SMU = Social media usage. GAM = Gaming Frequency. INT = Internalising symptoms. T1, 2, 3 = Time 1, 2, 3.

* p < .05, ** p < .01, *** p < .001

**Table S11**

*Analysis Incorporating Active Social Media Usage in the Model*

|  |  | Girls | | | |  | Boys | | | |
| --- | --- | --- | --- | --- | --- | --- | --- | --- | --- | --- |
| Time | Path | *b* | *p* | *CI* | *beta* |  | *b* | *p* | *CI* | *beta* |
|  |  | Cross-lagged Pathways | | | | | | | | |
| T1 to T2 | GAM → INT | 0.007 | 0.376 | [-0.009, 0.024] | 0.022 |  | 0 | 0.999 | [-0.025, 0.025] | 0 |
| T2 to T3 | GAM → INT | 0.007 | 0.376 | [-0.009, 0.024] | 0.023 |  | -0.017 | 0.264 | [-0.046, 0.013] | -0.032 |
| T1 to T2 | GAM → SMU | 0 | 0.99 | [-0.069, 0.070] | 0 |  | -0.069 | 0.099 | [-0.151, 0.013] | -0.031 |
| T2 to T3 | GAM → SMU | -0.068** | 0.006 | [-0.117, -0.019] | -0.059 |  | -0.069 | 0.099 | [-0.151, 0.013] | -0.036 |
| T1 to T2 | INT → GAM | 0.037 | 0.651 | [-0.123, 0.197] | 0.011 |  | -0.109 | 0.22 | [-0.283, 0.065] | -0.045 |
| T2 to T3 | INT → GAM | 0.037 | 0.651 | [-0.123, 0.197] | 0.012 |  | -0.234** | 0.003 | [-0.388, -0.081] | -0.104 |
| T1 to T2 | INT → SMU | -0.084 | 0.368 | [-0.268, 0.099] | -0.021 |  | 0.064 | 0.581 | [-0.165, 0.293] | 0.013 |
| T2 to T3 | INT → SMU | -0.084 | 0.368 | [-0.268, 0.099] | -0.024 |  | 0.064 | 0.581 | [-0.165, 0.293] | 0.016 |
| T1 to T2 | SMU → GAM | -0.032* | 0.046 | [-0.063, -0.001] | -0.041 |  | -0.018 | 0.09 | [-0.038, 0.003] | -0.037 |
| T2 to T3 | SMU → GAM | -0.032* | 0.046 | [-0.063, -0.001] | -0.039 |  | -0.018 | 0.09 | [-0.038, 0.003] | -0.033 |
| T1 to T2 | SMU → INT | -0.008 | 0.098 | [-0.017, 0.001] | -0.031 |  | 0.007 | 0.173 | [-0.003, 0.017] | 0.031 |
| T2 to T3 | SMU → INT | -0.008 | 0.098 | [-0.017, 0.001] | -0.029 |  | 0.007 | 0.305 | [-0.006, 0.019] | 0.025 |
|  |  | Autoregressive Effects | | | | | | | | |
| T1 to T2 | GAM → GAM | 0.122*** | 0 | [0.056, 0.188] | 0.116 |  | 0.137*** | 0.001 | [0.058, 0.216] | 0.122 |
| T2 to T3 | GAM → GAM | 0.182*** | 0 | [0.125, 0.240] | 0.18 |  | 0.137*** | 0.001 | [0.058, 0.216] | 0.127 |
| T1 to T2 | INT → INT | 0.367*** | 0 | [0.284, 0.451] | 0.354 |  | 0.332*** | 0 | [0.226, 0.438] | 0.291 |
| T2 to T3 | INT → INT | 0.272*** | 0 | [0.185, 0.360] | 0.267 |  | 0.250*** | 0 | [0.155, 0.346] | 0.228 |
| T1 to T2 | SMU → SMU | 0.166*** | 0 | [0.109, 0.223] | 0.173 |  | 0.145*** | 0 | [0.090, 0.199] | 0.15 |
| T2 to T3 | SMU → SMU | 0.152*** | 0 | [0.085, 0.220] | 0.166 |  | 0.110*** | 0 | [0.058, 0.162] | 0.114 |

*Note.* SMU = Social media usage. GAM = Gaming Frequency. INT = Internalising symptoms. T1, 2, 3 = Time 1, 2, 3.

* p < .05, ** p < .01, *** p < .001

**Table S12**

*Analysis Incorporating Passive Social Media Usage in the Model*

|  |  | Girls | | | |  | Boys | | | |
| --- | --- | --- | --- | --- | --- | --- | --- | --- | --- | --- |
| Time | Path | *b* | *p* | *CI* | *beta* |  | *b* | *p* | *CI* | *beta* |
|  |  | Cross-lagged Pathways | | | | | | | | |
| T1 to T2 | GAM → INT | 0.006 | 0.451 | [-0.010, 0.023] | 0.019 |  | 0.004 | 0.767 | [-0.021, 0.029] | 0.007 |
| T2 to T3 | GAM → INT | 0.006 | 0.451 | [-0.010, 0.023] | 0.019 |  | -0.012 | 0.396 | [-0.041, 0.016] | -0.024 |
| T1 to T2 | GAM → SMU | -0.036 | 0.282 | [-0.102, 0.030] | -0.027 |  | 0.107* | 0.01 | [0.025, 0.189] | 0.048 |
| T2 to T3 | GAM → SMU | -0.058 | 0.061 | [-0.118, 0.003] | -0.048 |  | 0.107* | 0.01 | [0.025, 0.189] | 0.052 |
| T1 to T2 | INT → GAM | 0.027 | 0.747 | [-0.138, 0.192] | 0.008 |  | -0.109 | 0.216 | [-0.282, 0.064] | -0.045 |
| T2 to T3 | INT → GAM | 0.027 | 0.747 | [-0.138, 0.192] | 0.009 |  | -0.224** | 0.004 | [-0.376, -0.071] | -0.099 |
| T1 to T2 | INT → SMU | 0.172 | 0.063 | [-0.009, 0.353] | 0.042 |  | 0.061 | 0.585 | [-0.159, 0.281] | 0.013 |
| T2 to T3 | INT → SMU | 0.172 | 0.063 | [-0.009, 0.353] | 0.046 |  | 0.061 | 0.585 | [-0.159, 0.281] | 0.014 |
| T1 to T2 | SMU → GAM | -0.005 | 0.744 | [-0.037, 0.027] | -0.007 |  | 0 | 0.986 | [-0.017, 0.018] | 0 |
| T2 to T3 | SMU → GAM | -0.005 | 0.744 | [-0.037, 0.027] | -0.007 |  | 0 | 0.986 | [-0.017, 0.018] | 0 |
| T1 to T2 | SMU → INT | 0.005 | 0.336 | [-0.005, 0.016] | 0.021 |  | -0.019*** | 0.001 | [-0.030, -0.008] | -0.083 |
| T2 to T3 | SMU → INT | 0.005 | 0.336 | [-0.005, 0.016] | 0.02 |  | -0.007 | 0.274 | [-0.020, 0.006] | -0.027 |
|  |  | Autoregressive Effects | | | | | | | | |
| T1 to T2 | GAM → GAM | 0.122*** | 0 | [0.056, 0.187] | 0.115 |  | 0.138*** | 0 | [0.060, 0.215] | 0.123 |
| T2 to T3 | GAM → GAM | 0.183*** | 0 | [0.125, 0.240] | 0.181 |  | 0.138*** | 0 | [0.060, 0.215] | 0.129 |
| T1 to T2 | INT → INT | 0.355*** | 0 | [0.268, 0.441] | 0.341 |  | 0.336*** | 0 | [0.230, 0.442] | 0.295 |
| T2 to T3 | INT → INT | 0.267*** | 0 | [0.178, 0.356] | 0.261 |  | 0.256*** | 0 | [0.161, 0.350] | 0.233 |
| T1 to T2 | SMU → SMU | 0.131*** | 0 | [0.074, 0.188] | 0.136 |  | 0.060* | 0.042 | [0.002, 0.118] | 0.061 |
| T2 to T3 | SMU → SMU | 0.099** | 0.003 | [0.034, 0.165] | 0.105 |  | 0.121*** | 0 | [0.061, 0.181] | 0.119 |

*Note.* SMU = Social media usage. GAM = Gaming Frequency. INT = Internalising symptoms. T1, 2, 3 = Time 1, 2, 3.

* p < .05, ** p < .01, *** p < .001

**Table S13**

*Analysis Using a Sample of Participants with at Least Two Waves of Data*

|  |  | Girls | | | |  | Boys | | | |
| --- | --- | --- | --- | --- | --- | --- | --- | --- | --- | --- |
| Time | Path | *b* | *p* | *CI* | *beta* |  | *b* | *p* | *CI* | *beta* |
|  |  | Cross-lagged Pathways | | | | | | | | |
| T1 to T2 | GAM → INT | 0.006 | 0.465 | [-0.010, 0.022] | 0.018 |  | 0.001 | 0.939 | [-0.024, 0.026] | 0.002 |
| T2 to T3 | GAM → INT | 0.006 | 0.465 | [-0.010, 0.022] | 0.018 |  | -0.012 | 0.422 | [-0.040, 0.017] | -0.022 |
| T1 to T2 | GAM → SMU | -0.049 | 0.363 | [-0.155, 0.057] | -0.027 |  | 0.041 | 0.527 | [-0.086, 0.169] | 0.013 |
| T2 to T3 | GAM → SMU | -0.142*** | 0.001 | [-0.225, -0.059] | -0.087 |  | 0.041 | 0.527 | [-0.086, 0.169] | 0.015 |
| T1 to T2 | INT → GAM | 0.036 | 0.657 | [-0.125, 0.198] | 0.011 |  | -0.107 | 0.205 | [-0.273, 0.059] | -0.044 |
| T2 to T3 | INT → GAM | 0.036 | 0.657 | [-0.125, 0.198] | 0.012 |  | -0.204** | 0.007 | [-0.351, -0.057] | -0.091 |
| T1 to T2 | INT → SMU | 0.002 | 0.988 | [-0.257, 0.261] | 0 |  | 0.162 | 0.351 | [-0.178, 0.502] | 0.024 |
| T2 to T3 | INT → SMU | 0.002 | 0.988 | [-0.257, 0.261] | 0 |  | 0.162 | 0.351 | [-0.178, 0.502] | 0.029 |
| T1 to T2 | SMU → GAM | -0.022 | 0.132 | [-0.050, 0.007] | -0.041 |  | -0.009 | 0.249 | [-0.023, 0.006] | -0.026 |
| T2 to T3 | SMU → GAM | -0.022 | 0.132 | [-0.050, 0.007] | -0.037 |  | -0.009 | 0.249 | [-0.023, 0.006] | -0.023 |
| T1 to T2 | SMU → INT | -0.002 | 0.673 | [-0.009, 0.006] | -0.009 |  | -0.005 | 0.138 | [-0.012, 0.002] | -0.034 |
| T2 to T3 | SMU → INT | -0.002 | 0.673 | [-0.009, 0.006] | -0.008 |  | 0.001 | 0.914 | [-0.009, 0.010] | 0.003 |
|  |  | Autoregressive Effects | | | | | | | | |
| T1 to T2 | GAM → GAM | 0.123*** | 0 | [0.057, 0.188] | 0.117 |  | 0.140*** | 0 | [0.067, 0.213] | 0.123 |
| T2 to T3 | GAM → GAM | 0.181*** | 0 | [0.124, 0.238] | 0.179 |  | 0.140*** | 0 | [0.067, 0.213] | 0.131 |
| T1 to T2 | INT → INT | 0.372*** | 0 | [0.287, 0.457] | 0.35 |  | 0.335*** | 0 | [0.228, 0.443] | 0.287 |
| T2 to T3 | INT → INT | 0.282*** | 0 | [0.198, 0.366] | 0.277 |  | 0.257*** | 0 | [0.165, 0.350] | 0.234 |
| T1 to T2 | SMU → SMU | 0.233*** | 0 | [0.174, 0.291] | 0.257 |  | 0.139*** | 0 | [0.091, 0.188] | 0.151 |
| T2 to T3 | SMU → SMU | 0.184*** | 0 | [0.102, 0.266] | 0.194 |  | 0.115*** | 0 | [0.054, 0.175] | 0.119 |

*Note.* SMU = Social media usage. GAM = Gaming Frequency. INT = Internalising symptoms. T1, 2, 3 = Time 1, 2, 3.

* p < .05, ** p < .01, *** p < .001

**Table S14**

*Analysis Using a Sample of Participants with at Least Three Waves of Data*

|  |  | Girls | | | |  | Boys | | | |
| --- | --- | --- | --- | --- | --- | --- | --- | --- | --- | --- |
| Time | Path | *b* | *p* | *CI* | *beta* |  | *b* | *p* | *CI* | *beta* |
|  |  | Cross-lagged Pathways | | | | | | | | |
| T1 to T2 | GAM → INT | 0.008 | 0.426 | [-0.011, 0.027] | 0.023 |  | -0.001 | 0.966 | [-0.029, 0.028] | -0.001 |
| T2 to T3 | GAM → INT | 0.008 | 0.426 | [-0.011, 0.027] | 0.024 |  | -0.007 | 0.674 | [-0.042, 0.027] | -0.014 |
| T1 to T2 | GAM → SMU | -0.057 | 0.356 | [-0.178, 0.064] | -0.032 |  | 0.047 | 0.52 | [-0.096, 0.190] | 0.014 |
| T2 to T3 | GAM → SMU | -0.123** | 0.008 | [-0.214, -0.032] | -0.077 |  | 0.047 | 0.52 | [-0.096, 0.190] | 0.017 |
| T1 to T2 | INT → GAM | 0.007 | 0.948 | [-0.189, 0.203] | 0.002 |  | -0.102 | 0.321 | [-0.303, 0.099] | -0.044 |
| T2 to T3 | INT → GAM | 0.007 | 0.948 | [-0.189, 0.203] | 0.002 |  | -0.189* | 0.02 | [-0.348, -0.030] | -0.086 |
| T1 to T2 | INT → SMU | -0.085 | 0.579 | [-0.384, 0.215] | -0.015 |  | 0.172 | 0.314 | [-0.163, 0.507] | 0.026 |
| T2 to T3 | INT → SMU | -0.085 | 0.579 | [-0.384, 0.215] | -0.017 |  | 0.172 | 0.314 | [-0.163, 0.507] | 0.03 |
| T1 to T2 | SMU → GAM | -0.026 | 0.091 | [-0.056, 0.004] | -0.049 |  | -0.003 | 0.695 | [-0.020, 0.013] | -0.01 |
| T2 to T3 | SMU → GAM | -0.026 | 0.091 | [-0.056, 0.004] | -0.043 |  | -0.003 | 0.695 | [-0.020, 0.013] | -0.009 |
| T1 to T2 | SMU → INT | -0.008 | 0.059 | [-0.017, 0.000] | -0.047 |  | -0.006 | 0.139 | [-0.014, 0.002] | -0.038 |
| T2 to T3 | SMU → INT | -0.008 | 0.059 | [-0.017, 0.000] | -0.042 |  | 0.001 | 0.793 | [-0.010, 0.012] | 0.008 |
|  |  | Autoregressive Effects | | | | | | | | |
| T1 to T2 | GAM → GAM | 0.158*** | 0 | [0.082, 0.234] | 0.152 |  | 0.124** | 0.004 | [0.039, 0.209] | 0.109 |
| T2 to T3 | GAM → GAM | 0.169*** | 0 | [0.102, 0.237] | 0.167 |  | 0.124** | 0.004 | [0.039, 0.209] | 0.113 |
| T1 to T2 | INT → INT | 0.388*** | 0 | [0.306, 0.470] | 0.363 |  | 0.301*** | 0 | [0.183, 0.420] | 0.263 |
| T2 to T3 | INT → INT | 0.302*** | 0 | [0.212, 0.391] | 0.296 |  | 0.241*** | 0 | [0.143, 0.340] | 0.22 |
| T1 to T2 | SMU → SMU | 0.219*** | 0 | [0.151, 0.286] | 0.243 |  | 0.143*** | 0 | [0.091, 0.196] | 0.155 |
| T2 to T3 | SMU → SMU | 0.162*** | 0 | [0.071, 0.252] | 0.171 |  | 0.122*** | 0 | [0.058, 0.185] | 0.126 |

*Note.* SMU = Social media usage. GAM = Gaming Frequency. INT = Internalising symptoms. T1, 2, 3 = Time 1, 2, 3.

* p < .05, ** p < .01, *** p < .001

**Table S15**

*Analysis Using the Original Game Variable with Six Responses*

|  |  | Girls | | | |  | Boys | | | |
| --- | --- | --- | --- | --- | --- | --- | --- | --- | --- | --- |
| Time | Path | *b* | *p* | *CI* | *beta* |  | *b* | *p* | *CI* | *beta* |
|  |  | Cross-lagged Pathways | | | | | | | | |
| T1 to T2 | GAM → INT | 0.006 | 0.3 | [-0.005, 0.017] | 0.026 |  | -0.004 | 0.618 | [-0.017, 0.010] | -0.011 |
| T2 to T3 | GAM → INT | 0.006 | 0.3 | [-0.005, 0.017] | 0.026 |  | -0.004 | 0.618 | [-0.017, 0.010] | -0.011 |
| T1 to T2 | GAM → SMU | -0.013 | 0.748 | [-0.095, 0.068] | -0.011 |  | 0.035 | 0.39 | [-0.045, 0.116] | 0.018 |
| T2 to T3 | GAM → SMU | -0.092** | 0.004 | [-0.154, -0.029] | -0.079 |  | 0.035 | 0.39 | [-0.045, 0.116] | 0.021 |
| T1 to T2 | INT → GAM | 0.091 | 0.428 | [-0.134, 0.316] | 0.02 |  | -0.077 | 0.558 | [-0.333, 0.180] | -0.02 |
| T2 to T3 | INT → GAM | 0.091 | 0.428 | [-0.134, 0.316] | 0.021 |  | -0.302* | 0.013 | [-0.541, -0.064] | -0.086 |
| T1 to T2 | INT → SMU | 0.023 | 0.858 | [-0.234, 0.280] | 0.004 |  | 0.147 | 0.405 | [-0.199, 0.493] | 0.022 |
| T2 to T3 | INT → SMU | 0.023 | 0.858 | [-0.234, 0.280] | 0.004 |  | 0.147 | 0.405 | [-0.199, 0.493] | 0.026 |
| T1 to T2 | SMU → GAM | -0.039 | 0.07 | [-0.082, 0.003] | -0.053 |  | -0.018 | 0.107 | [-0.040, 0.004] | -0.035 |
| T2 to T3 | SMU → GAM | -0.039 | 0.07 | [-0.082, 0.003] | -0.048 |  | -0.018 | 0.107 | [-0.040, 0.004] | -0.03 |
| T1 to T2 | SMU → INT | 0 | 0.985 | [-0.007, 0.008] | 0 |  | -0.005 | 0.147 | [-0.013, 0.002] | -0.034 |
| T2 to T3 | SMU → INT | 0 | 0.985 | [-0.007, 0.008] | 0 |  | 0 | 0.946 | [-0.010, 0.010] | -0.002 |
|  |  | Autoregressive Effects | | | | | | | | |
| T1 to T2 | GAM → GAM | 0.139*** | 0 | [0.073, 0.205] | 0.133 |  | 0.180*** | 0 | [0.107, 0.254] | 0.163 |
| T2 to T3 | GAM → GAM | 0.182*** | 0 | [0.120, 0.244] | 0.184 |  | 0.180*** | 0 | [0.107, 0.254] | 0.17 |
| T1 to T2 | INT → INT | 0.301*** | 0 | [0.224, 0.378] | 0.292 |  | 0.334*** | 0 | [0.228, 0.440] | 0.293 |
| T2 to T3 | INT → INT | 0.301*** | 0 | [0.224, 0.378] | 0.285 |  | 0.254*** | 0 | [0.160, 0.347] | 0.231 |
| T1 to T2 | SMU → SMU | 0.232*** | 0 | [0.172, 0.292] | 0.256 |  | 0.137*** | 0 | [0.089, 0.185] | 0.15 |
| T2 to T3 | SMU → SMU | 0.190*** | 0 | [0.108, 0.272] | 0.199 |  | 0.106*** | 0.001 | [0.044, 0.169] | 0.11 |

*Note.* SMU = Social media usage. GAM = Gaming Frequency. INT = Internalising symptoms. T1, 2, 3 = Time 1, 2, 3.

* p < .05, ** p < .01, *** p < .001

**Table S16.**

*Results of the Multigroup RI-CLPM Using Multiple Imputation for Participants with at Least Two Survey Waves*

|  |  | *Girl* | |  | *Boy* | |
| --- | --- | --- | --- | --- | --- | --- |
| Time | Path | *b* | *p* |  | *b* | *p* |
|  |  | Cross-lagged Pathways | | | | |
| T1 to T2 | GAM → INT | 0.01 | 0.381 |  | 0 | 0.876 |
| T2 to T3 | GAM → INT | 0.01 | 0.381 |  | -0.01 | 0.453 |
| T1 to T2 | GAM → SMU | -0.04 | 0.306 |  | 0.04 | 0.531 |
| T2 to T3 | GAM → SMU | -0.13** | 0.002 |  | 0.04 | 0.531 |
| T1 to T2 | INT → GAM | 0.03 | 0.747 |  | -0.11 | 0.155 |
| T2 to T3 | INT → GAM | 0.03 | 0.747 |  | -0.20** | 0.002 |
| T1 to T2 | INT → SMU | 0 | 0.983 |  | 0.16 | 0.31 |
| T2 to T3 | INT → SMU | 0 | 0.983 |  | 0.16 | 0.31 |
| T1 to T2 | SMU → GAM | -0.02 | 0.132 |  | -0.01 | 0.352 |
| T2 to T3 | SMU → GAM | -0.02 | 0.132 |  | -0.01 | 0.352 |
| T1 to T2 | SMU → INT | 0 | 0.761 |  | -0.01 | 0.18 |
| T2 to T3 | SMU → INT | 0 | 0.761 |  | 0 | 0.891 |
|  |  | Autoregressive Effects | | | | |
| T1 to T2 | GAM → GAM | 0.13*** | < .001 |  | 0.14*** | < .001 |
| T2 to T3 | GAM → GAM | 0.19*** | < .001 |  | 0.14*** | < .001 |
| T1 to T2 | INT → INT | 0.38*** | < .001 |  | 0.38*** | < .001 |
| T2 to T3 | INT → INT | 0.29*** | < .001 |  | 0.30*** | < .001 |
| T1 to T2 | SMU → SMU | 0.23*** | < .001 |  | 0.14*** | < .001 |
| T2 to T3 | SMU → SMU | 0.18*** | < .001 |  | 0.11*** | < .001 |

*Note.* SMU = Social media usage. GAM = Gaming Frequency. INT = Internalising symptoms. T1, 2, 3 = Time 1, 2, 3.

*p < .05, **p <.01, ***p <.001

**Table S17.**

*Results of the Multigroup RI-CLPM Using Multiple Imputation for Participants with at Least One Survey Waves*

|  |  | *Girl* | |  | *Boy* | |
| --- | --- | --- | --- | --- | --- | --- |
| Path | Time | *b* | *p* |  | *b* | *p* |
|  |  | Cross-lagged Pathways | | | | |
| T1 to T2 | GAM → INT | 0 | 0.465 |  | 0 | 0.863 |
| T2 to T3 | GAM → INT | 0 | 0.465 |  | -0.01 | 0.272 |
| T1 to T2 | GAM → SMU | -0.05 | 0.201 |  | 0.03 | 0.621 |
| T2 to T3 | GAM → SMU | -0.13*** | < .001 |  | 0.03 | 0.621 |
| T1 to T2 | INT → GAM | 0.03 | 0.753 |  | -0.1 | 0.238 |
| T2 to T3 | INT → GAM | 0.03 | 0.753 |  | -0.22*** | < .001 |
| T1 to T2 | INT → SMU | 0.04 | 0.761 |  | 0.11 | 0.505 |
| T2 to T3 | INT → SMU | 0.04 | 0.761 |  | 0.11 | 0.505 |
| T1 to T2 | SMU → GAM | -0.02 | 0.125 |  | -0.01 | 0.254 |
| T2 to T3 | SMU → GAM | -0.02 | 0.125 |  | -0.01 | 0.254 |
| T1 to T2 | SMU → INT | 0 | 0.741 |  | 0 | 0.18 |
| T2 to T3 | SMU → INT | 0 | 0.741 |  | 0 | 0.565 |
|  |  | Autoregressive Effects | | | | |
| T1 to T2 | GAM → GAM | 0.14*** | < .001 |  | 0.13*** | < .001 |
| T2 to T3 | GAM → GAM | 0.19*** | < .001 |  | 0.13*** | < .001 |
| T1 to T2 | INT → INT | 0.38*** | < .001 |  | 0.41*** | < .001 |
| T2 to T3 | INT → INT | 0.29*** | < .001 |  | 0.32*** | < .001 |
| T1 to T2 | SMU → SMU | 0.23*** | < .001 |  | 0.14*** | < .001 |
| T2 to T3 | SMU → SMU | 0.19*** | < .001 |  | 0.10*** | < .001 |

*Note.* SMU = Social media usage. GAM = Gaming Frequency. INT = Internalising symptoms. T1, 2, 3 = Time 1, 2, 3.

* p < .05, ** p < .01, *** p < .001

**Reference:**

Black, L., Humphrey, N., Panayiotou, M., & Marquez, J. (2024). Mental Health and Well-being Measures for Mean Comparison and Screening in Adolescents: An Assessment of Unidimensionality and Sex and Age Measurement Invariance. *Assessment*, 31(2), 219-236. <https://doi.org/10.1177/10731911231158623>

Deighton, J., Tymms, P., Vostanis, P., Belsky, J., Fonagy, P., Brown, A., Martin, A., Patalay, P., & Wolpert, M. (2013). The Development of a School-Based Measure of Child Mental Health. *Journal of Psychoeducational Assessment,* 31(3), 247-257. <https://doi.org/10.1177/0734282912465570>

Enders, C. K. (2022). *Applied missing data analysis*. Second Edition. Guilford Publications.

Hall, R. J., Snell, A. F., & Foust, M. S. (1999). Item Parceling Strategies in SEM: Investigating the Subtle Effects of Unmodeled Secondary Constructs. *Organizational Research Methods*, *2*(3), 233-256. https://doi.org/10.1177/109442819923002

Little, T. D., Rioux, C., Odejimi, O. A., & Stickley, Z. L. (2022). *Parceling in Structural Equation Modeling: A Comprehensive Introduction for Developmental Scientists.* Cambridge: Cambridge University Press.

McNeish, D., & Wolf, M. G. (2023). Dynamic fit index cutoffs for confirmatory factor analysis models. *Psychological Methods*, 28(1), 61–88. https://doi.org/10.1037/met0000425
